# Association analysis for disease resistance to *Fusarium oxysporum* in cape gooseberry (*Physalis peruviana* L)

**DOI:** 10.1186/s12864-016-2568-7

**Published:** 2016-03-18

**Authors:** Jaime A. Osorio-Guarín, Felix E. Enciso-Rodríguez, Carolina González, Noé Fernández-Pozo, Lukas A. Mueller, Luz Stella Barrero

**Affiliations:** Tibaitatá Research Center, Colombian Corporation for Agricultural Research, Corpoica, Km 14 vía Mosquera, Bogotá, Colombia; Agrobiodiversity Department, National Direction of Research and Development, Corpoica, Km 14 vía Mosquera, Bogotá, Colombia; Boyce Thompson Institute for Plant Research, Ithaca, NY USA

**Keywords:** Association mapping, Cape gooseberry, *Fusarium oxysporum*, Genotyping by sequencing

## Abstract

**Background:**

Vascular wilt caused by *Fusarium oxysporum* is the most important disease in cape gooseberry (*Physalis peruviana* L.) in Colombia. The development of resistant cultivars is considered one of the most cost-effective means to reduce the impact of this disease. In order to do so, it is necessary to provide breeders with molecular markers and promising germplasm for introgression of different resistance loci as part of breeding schemes. Here we described an association mapping study in cape gooseberry with the goal to: (i) select promising materials for use in plant breeding and (ii) identify SNPs associated with the cape gooseberry resistance response to the *F. oxysporum* pathogen under greenhouse conditions, as potential markers for cape gooseberry breeding.

**Results:**

We found a total of 21 accessions with different resistance responses within a diversity panel of 100 cape gooseberry accessions. A total of 60,663 SNPs were also identified within the same panel by means of GBS (Genotyping By Sequencing). Model-based population structure and neighbor-joining analyses showed three populations comprising the cape gooseberry panel. After correction for population structure and kinship, we identified SNPs markers associated with the resistance response against *F. oxysporum*. The identification of markers was based on common tags using the reference genomes of tomato and potato as well as the root/stem transcriptome of cape gooseberry. By comparing their location with the tomato genome, 16 SNPs were found in genes involved in defense/resistance response to pathogens, likewise when compared with the genome of potato, 12 markers were related.

**Conclusions:**

The work presented herein provides the first association mapping study in cape gooseberry showing both the identification of promising accessions with resistance response phenotypes and the identification of a set of SNP markers mapped to defense/resistance response genes of reference genomes. Thus, the work also provides new knowledge on candidate genes involved in the *P. peruviana* – *F. oxysporum* pathosystem as a foundation for further validation in marker-assisted selection. The results have important implications for conservation and breeding strategies in cape gooseberry.

**Electronic supplementary material:**

The online version of this article (doi:10.1186/s12864-016-2568-7) contains supplementary material, which is available to authorized users.

## Background

The cape gooseberry (*Physalis peruviana* L.) is a species within the *Solanaceae* family widely used for medicinal and commercial purposes. It is native in the Andean region, primarily Colombia, Peru and Ecuador [1]. It is the second most important exported fruit in Colombia, which is the world’s top producer, with total sales of $ 27.6 million for 2013 [2]. The cape gooseberry production has suffered a major decline in Colombia, from 1087 ha with a yield of 17.8 t in 2009, to 749 ha with a yield of 15 t in 2013 [2, 3]. One of the major causes for this decline is the vascular wilt disease caused by the soil-borne fungus *Fusarium oxysporum*, which is an important phytosanitary problem that is still unmanageable [4].

The fungus *F. oxysporum* is the causal agent of vascular wilt in several species of plants, such as the cucumber (*Cucumis sativus L.*), carnation (*Dianthus caryophyllus L.*), tomato (*Solanum lycopersicum L.*) and potato (*Solanum tuberosum L.*) causing yield losses between 20 and 70 % [5, 6]. The management of this disease is hampered by the pathogen adaptation including resistance to the commercial fungicides, and its long term survival in the soil due to production of resistant structures called chlamydospores [7].

Several disease control methods have been attempted to reduce the incidence of vascular wilt. Crop rotation does not offer an effective solution because of the presence of chlamydospores in the soil. Another option is soil fumigation; however, it is not a long-term solution because recolonization frequently occurs. Finally, soil treatments with compost or enriched compost using selected microorganisms represent a possible management alternative [8, 9]. However, the development of resistant cultivars is one of the most promising alternatives for reducing the negative impact of *Fusarium* infection. This alternative will reduce the dependency on chemical protection, resulting in safer, more affordable and less environmentally detrimental cultivation of these crops [10]. Successful cases of the development of resistant varieties to *F. oxysporum* have been reported in various species including lettuce [11], cucumber [12], tomato [13], among others.

The development of resistant cultivars can-be-accelerated by using markers associated with resistance/defense Quantitative Trait Loci (QTLs). The continuing advances in QTL identification including Association Mapping (AM) studies are accelerating the identification of genes related with disease resistance, like the loci *I-1*, *I-2* and *I-3* in chromosomes 11 and 7 from tomato which confer resistance to *F. oxysporum* f. sp. *lycopersici* [13, 14]. More recently, Genome-wide Association Studies (GWAS) in conjunction with Genomic Selection (GS) has shown to provide an effective tool for increasing the efficiency of crop breeding [15].

Single Nucleotide Polymorphisms (SNPs) are usually the markers of choice for QTL identification as well as for studies of genetic diversity and population structure required for association studies [16]. Genotyping By Sequencing (GBS), a highly multiplexed method based on reducing genome complexity through methylation-sensitive restriction enzymes, uses next generation sequencing technologies to identify large sets of SNPs [17]. GBS can be suitable for species with high diversity and large genomes even without the need of a reference genome [18]. The latter suggests that GBS can be appropriate for orphan species such as cape gooseberry. This approach has been successfully used, for example, in a study conducted by Lambel et al. [19], where a major QTL associated with resistance to *F. oxysporum* f. sp *niveum* race 1 on chromosome 1 of the watermelon genetic map was identified. In addition, a minor QTLs were identified on chromosomes 1, 3, 4, 9 and 10.

The present study aims to: 1) assess the resistance phenotype of a diversity panel of 100 cape gooseberry accessions to *F. oxysporum* in greenhouse conditions; 2) identify marker-trait associations for the resistance response to *F. oxysporum* based on GBS as a foundation for future GWAS/GS studies in cape gooseberry. The SNPs associated with resistance/defense regions will increase the knowledge of mechanisms underlying disease resistance, providing tools for Marker-Assisted Selection (MAS) or other new molecular selection methods to accumulate desirable genes in breeding programs.

## Methods

### Mapping population

The cape gooseberry association mapping population used in this study comprised a diversity panel of 100 accessions from the germplasm collection managed by the Colombian Corporation for Agricultural Research (CORPOICA) (Additional file 1: Table S1). This population was composed of wild or cultivated accessions that were selected based on the following criteria: a) presence of passport data, b) representativeness of the main producing geographic areas in Colombia, c) wide geographic distribution, d) genetic diversity based on molecular markers [20], (e) different resistance and susceptibility responses against *F. oxysporum* under greenhouse conditions based on an initial screening conducted by Enciso-Rodríguez et al. [21]. We selected some of these accessions with the aim of covering the extremes of the distribution of the phenotypic variance. In addition, nine accessions obtained as double haploids (DH) derived from cultivated germplasm used for breeding purposes [22], were also included within the panel group.

### Pathogen isolation and inoculum preparation

The highly virulent monosporic strain of *F. oxysporum* (Map5) isolated from *P. peruviana* plants in field [21] and preserved in filter paper at −20 °C was used for inoculum preparation. The Map5 isolate was reactivated in Potato Dextrose Agar (PDA) medium for 10 days. A piece of 1 cm^2^ of agar with mycelium growth were cut and was grown in liquid Potato Dextrose Broth (PDB) for 8 days at 27 °C in constant shaking and then adjusted to a final concentration of 1×10^6^ conidia/ml according to Namiki et al. [23]. A volume of 100 mL of conidia suspension per each 900 g of soil was adjusted according with the methodology described by Moreno et al. [24], to inoculated by aspersion of the substrate (soil : peat : rice husk) mixed in a proportion 3: 1: 1.

### Evaluation of *Fusarium oxysporum* resistance

The plantlets of *P. peruviana* accessions were clonally multiplied *in vitro* from node cuttings*.* Once a pair of true leaves appeared and plantlets were 5 to 7 cm tall (3 months old), eight plants per accession were transplanted individually into plastic pots with 600 g of inoculated substrate and two plants were mock-inoculated as negative control. The screening for resistance to the pathogen was carried out in a greenhouse at 26 + 2 °C, with light/dark photoperiods of 12/12 h, with a relative humidity of 70–80 % in the Corpoica’s facilities in Mosquera, Colombia. The symptoms were scored 3 times a week over 47 days using a nine-grade severity scale, with 0 denoting high tolerance and nine high susceptibility. This scale was described by Enciso-Rodríguez et al. [21], using 15 genotypes from *P. peruviana* and related taxa inoculated with the Map5 *F. oxysporum* pathogenic strain. The percentage of incidence was calculated as the number of new cases of disease during specified period divided by size of population at start of period. The Area Under the Disease Progress Curve (AUDPC) based on the severity scale was calculated using the formula proposed by Shanner and Finner [25]. Accessions that displayed potential resistance response after the initial screening, were re-transplanted and re-inoculated, using the same procedure described above.

### Statistic analysis of phenotypic data

The phenotypic data were statistically analyzed through Shapiro-Wilks normality test using the software package SAS v9.1.3 (SAS Institute, Cary NC) [26] and normalized by implementing the Box-Cox transformation with the software STATISTICA v12.0 (Statsoft Inc., Tulsa, USA). In addition, a Ward algorithm conglomerate analysis from Principal Component Analysis (PCA), applying the PRINCOMP procedure in SAS v9.1.3, was used to obtain a dendrogram.

### Genotyping

Total DNA was isolated from young leaves collected from each accession using DNeasy Plant Mini Kit (QIAGEN, Germany) according to manufacturer’s instructions. The final elution volume was adjusted to 70 μl with TE solution buffer. Total DNA was quantified using λ *Hind*III size/mass (Invitrogen) and the quality was inspected using restriction enzyme digestions with *Hind*III enzyme, and visualized by electrophoresis using 2 % agarose gels. The GBS libraries were constructed at Cornell Genomic Diversity Facility (USA), library duplicates were used as technical replicates in 95-plex using the restriction enzyme *Apek*I (GCWGC) and barcoded adapters were ligated to individual samples. Genotyping was performed following the GBS protocol by Elshire et al. [17] and multiplexing on a single lane of Illumina HiSeq 2000.

### SNP discovery and data processing

FASTQ files obtained from sequencing were processed using the GBS pipeline implemented on TASSEL standalone v4.3.5 [27, 28]. The pipeline’s first step is the multiplexing using the barcode adapter “key file”. Then, identical aligned reads were clustered into tags (reads consisting of a cut site remnant and additional sequence of 64 bp), and then the reads were aligned using BOWTIE2 [29] to the tomato and potato reference genomes [30, 31], as well as to the cape gooseberry root/stem transcriptome (NCBI Bioproject ID No. PRJNA67621), separately. The tags that were aligned to each one of the reference genomes/transcriptome, and were filtered into common tags between the two references genomes and the reference transcriptome. The parameters used for high quality SNP detection for common tags were: minimum allele frequency of 0.01 (overall), minimum locus coverage^1^ (mnLCov) of 0.9, minimum site coverage^2^ (mnScov) of 0.7, minimum taxon coverage^3^ (mnTCov) of 0.5. A filter of high linkage disequilibrium (hLD), was also used to filter the SNPs with significant threshold of *r*^2^ ≥ 0.1.

### Genetic diversity

Population estimates of genetic diversity were analyzed using the SNPs of cape gooseberry transcriptome and then separately for common tags. The program POWERMARKER v3.25 [32] was used to calculate allele frequencies, observed heterozygosity (*Ho*), expected heterozygosity (*He*) and Polymorphism Information Content (PIC).

### Population structure

Distance matrix based on Identity By State (IBS) similarity, defined as the probability that alleles drawn at random from two individuals at the same locus are the same, was calculated from HapMap files using TASSEL v4.3.5 and the resulting matrix was clustered by the Neighbor-Joining algorithm, and visualized by FIGTREE v1.4.0 [33]. The SNP marker matrix used for PCA analysis was obtained from the Variant Call Format (VCF) files generated by TASSEL v4.3.5, using *gdsfmt* and *SNPRelate* [34] packages implemented on the statistical software R [35].

In order to estimate the number of sub-populations in the sampled plant accessions, a bayesian model clustering analysis was carried out on the best set of SNPs for optimizing the run, applying the admixture model for the ancestry of individuals using the software STRUCTURE v2.3.4 [36] with the following parameters: number of populations (K) set from 1 to 10, repeated 10 times, with a burn-in period of 50,000 iterations and 100,000 Markov Chain Monte Carlo (MCMC) repeats. The software CLUMPP v1.1.1 [37] was used to line up the cluster labels across runs and to estimate the degree of congruence between independent runs. Visualization of the results was done with DISTRUCT v1.1 [38]. The K optimum was evaluated by approaches described by Pritchard [36] and by Evanno et al. [39], using STRUCTURE HARVESTER [40].

### Linkage disequilibrium

The linkage disequilibrium (LD) between two SNPs was measured and visualized using r^2^ (*p*-value of ≤ 0.005) across each one of the reference genomes using the software TASSEL v4.3.5 with a sliding windows of 50 markers for exploring variation patterns of LD.

### Association analysis

The analysis was conducted using the Genome Association and Prediction Integrated Tool GAPIT [41], an R package [35]. Associations between polymorphisms and phenotypes were evaluated using the Mixed Linear Model (MLM) by incorporating phenotypic and genotypic data, population structure (Q) and kinship matrix (K), using the following formula: *y* = *Xa* + *Qb* + *Zu* + *e;* where *y* is vector for phenotypes; *a* is the vector of marker fixed effects, *b* is a vector of fixed effects, *u* is the vector of random effects (the kinship matrix), and *e* is the vector of residuals. *X* denotes the genotypes at the marker; *Q* is the Q-matrix and *Z* is an identity matrix [42]. The software STRUCTURE v2.3.4 had been used previously to determine the population structure of the diversity panel and the kinship matrix was calculated as described by Loiselle [43], and the False Discovery Rate (FDR), using the method proposed by Benjamini and Hochberg [44], was used for correcting spurious associations. The quantile-quantile plots (Q-Q plots) were constructed by ranking the sets of best association *p*-values and plotting them against the expected values, under the null hypothesis of no association.

The HapMap archives were used to infer the potential molecular function and the possible underlying biological process of the associated markers, using the Sol Genomics Network (SGN) (https://solgenomics.net/jbrowse/current/). For tomato (*Solanum lycopersicum*), the genomic annotation v2.3 realized by ITAG from SL2.40 genome construction was used. Additionally, for the potato genome (*Solanum tuberosum* group Phureja) the genomic annotation v3.4 realized by PGSC from DM3.40 genome construction was used.

## Results and discussion

### Evaluation of *Fusarium oxysporum* resistance

The first phenotypic response symptoms within the mapping population were observed at 14 days after inoculation. The mean of the susceptibility/resistance scale to measure vascular wilt, was six at 47 days after inoculation, with an incidence of 76 % (Additional file 2: Figure S1). AUDPC values ranged from 4.7 to 139.3 indicating a phenotypic variation for the disease severity within the mapping population. According to Simko & Piepho [45], the AUDPC value is effective for determining the progress of the disease, it gathers different observations during the epidemic and summarize all the values in a single one that reflects the severity of disease.

**Fig. 1:**
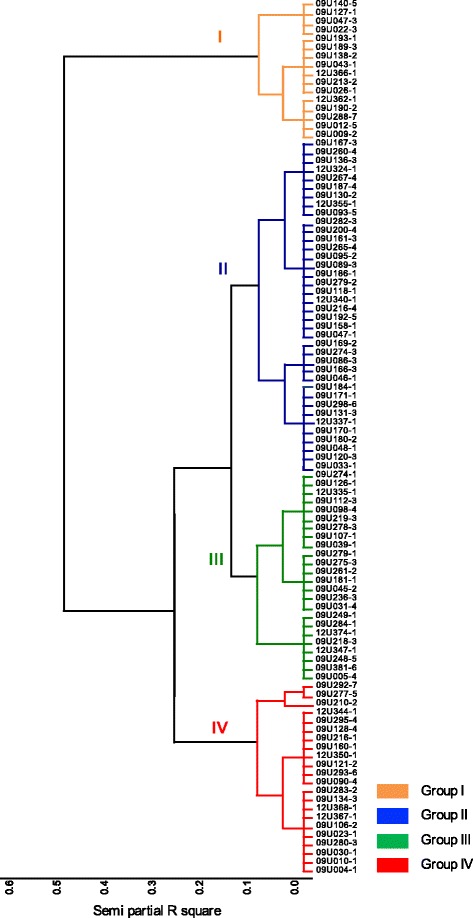
Ward algorithm conglomerate analysis of phenotypic data. Obtained from principal component analysis of severity and AUDPC variables

Based on conglomerate analysis using the Ward algorithm [46] (Fig. 1), the cape gooseberry’s accessions can be divided into four main groups. The first group (I) consisted of 13 wild accessions collected from five Colombian geographic departments (Antioquia, Boyacá, Cundinamarca, Norte de Santander, Valle del Cauca) and three accessions (09U288-7, 09U140-5 and 09U138-2) from the international repository of the USDA Plant Germplasm System. The second group (II) comprised 38 accessions collected mainly from six Colombian departments (Nariño, Antioquia, Cundinamarca, Boyacá, Santander, Valle del Cauca) as well as two accessions from international repositories. The third group (III) consisted of 24 cultivated accessions from four Colombian departments (Boyacá, Cundinamarca, Nariño, Boyacá). The fourth group (IV) consisted of 22 accessions, comprised mostly by DH accessions originating from *in vitro* culture of anthers [22].

The four groups reveal phenotypic variation for the resistance trait showing different levels of susceptibility/resistance responses to *F. oxysporum* that made the population suitable for association mapping*.* The first and the second group presented the highest values for resistance response with mean disease severity scales of 3.138 (ranged from 1.571 to 5) and of 5.725 (ranged from 3.571 to 7.200), respectively (Table 1). This result is consistent with the fact that these two groups are made up of wild-type germplasm. As reported by Chrispeels & Sadava [47] and Jiao et al. [48], wild type plant populations serves as a source of disease resistance traits; besides, study this populations to identifying SNPs associated with genes and predicting their function contribute to the breeding programs. One of the best-known cases is tomato, where race-specific R genes for resistance to *F. oxysporum* have been genetically mapped and introgressed into commercial cultivars from wild tomato species [49]. The third group had mean severity scales of 6.450 (ranged from 5.375 to 7.286, Table 1). This group was mainly represented by cultivated accessions used by farmers or commercial producers who usually select plants with good quality and yield. Cultivated germplasm do not necessarily have good resistance to pathogens [47]. The fourth group consisted of accessions highly susceptible to the pathogen (7.553 ranged from 5.750 to 9.0, Table 1). This group was represented by DH germplasm which may possess advantages to speed-up breeding processes towards fixing desirable alleles but at the same time can generate more susceptible homozygous materials which may accumulate deleterious recessive alleles, occurring in a process similar to inbreeding depression, possibly causing more susceptibility to pathogens [50]. Whether inbreeding depression occurs in cape gooseberry awaits further investigation.

**Table 1:** Statistics from ward algorithm clustering obtained from phenotypic data

Group	Variable	Mean	Minimum value	Maximum value	Coefficient of variation
1	Severity	3.138	1.571	5.000	29.697
	AUDPC	15.436	4.688	25.500	46.768
2	Severity	5.735	3.571	7.200	14.526
	AUDPC	38.205	16.800	66.812	27.253
3	Severity	6.450	5.375	7.286	7.747
	AUDPC	59.958	38.625	82.000	18.181
4	Severity	7.553	5.750	9.000	10.190
	AUDPC	83.932	63.500	139.312	21.406

*AUDPC* area under the disease progress curve

The Shapiro-Wilks test resulted in W-values of 0.946 (*p*-value = 0.0004) for severity with a negative tendency (−0.753) and 0.974 (*p*-value = 0.045) for AUDPC with a positive tendency (0.556), leading to rejection of the null hypothesis of normal distribution of the data. According to Mauricio [51], there is an implicit assumption that the trait values are normally distributed in quantitative trait loci (QTL) analysis. Violation of this assumption can severely affect the power of the analysis, incrementing the type I error. To resolve this difficulty, the data variance was stabilized using a Box-Cox transformation ensuring a normal distribution required for the association analysis and therefore improving the *p*-values of associated markers (Additional file 3: Figure S2).

### SNP discovery

GBS can be used for de novo discovery of SNPs, making it particularly powerful in germplasm collections and uncharacterized species [18]. GBS was used to genotype 100 accessions of the crop species *P. peruviana* using the *Apek*I restriction enzyme. This frequent*-*cutting enzyme has been used efficiently to produce high quality libraries in heterozygous species such as maize (*Zea mays*) because it generates large number of fragments necessary to cover a maximum number of recombination events [17]. Similarly, studies of GBS in soybean (*Glycine max* L.) validated *ApeK*I restriction enzyme as appropriate for plants because constructed libraries were rich in gene regions [52].

A total of 453,005,454 good quality reads were obtained from 505,347,672 raw reads that were generated from two Illumina HiSeq lanes. The TASSEL-GBS pipeline [28] clustered reads into 48,304,291 locus-specific tags with a mean depth per individual of 6.3 and a mean reads per accession of 2,359,883. Mapped tags per reference genome were 469,212 (1 % of the total tags) tags for tomato, 470,210 (1 % of the total tags) for potato and 416,989 (0,8 % of the total tags) for cape gooseberry and the common tags shared between three reference genomes were 120,124.

A set of 60,663 (19 % missing data) SNPs was identified using cape gooseberry root/stem transcriptome as reference using the default parameter filter implemented in the TASSEL-GBS pipeline. Other pipelines to discover SNPs in species without a reference genome have found 88,217 SNPs in switchgrass [53] and 45,117 SNPs in oat [54]. Those studies used the UNEAK pipeline or a combination of UNEAK and TASSEL. According to Glaubitz et al. [28], the TASSEL-GBS pipeline was designed for species with a reference genome; however, it is possible to use incomplete genome assemblies consisting of numerous contigs as a pseudo-reference. For tomato, potato and cape gooseberry as reference genomes/transcriptome we identified 1,739, 1,965 and 1,699 SNPs after filtering (2 % missing data) respectively using common tags. This approach was used in order to locate homologous sites between the cape gooseberry sequences and each one of the reference genomes, which represent the two closest high-quality sequenced and annotated genomes [30, 31]. Thus, we infer their putative relationship to genes related to pathogen resistance response, based on conservation of sequences and gene content in the Solanaceae family [55].

The percentage of missing data from GBS may be a serious problem, especially for association analysis. Better coverage can be achieved by two approaches: 1) repeated sequencing runs of the samples, although this solution increases costs, and quickly reaches a point where there is little reduction in missing data; and 2) imputation procedures based on identifying the most similar haplotype that can be used to supply some of those missing data [56]. The second approach is not possible with cape gooseberry because of the lack of a reference genome. In our case we reduced the missing from 19 to 2 % when each individual sample was sequenced by duplication. Therefore, the first approach was carried out for cape gooseberry in the present study.

### Genetic diversity, population structure, and linkage disequilibrium

The genetic analysis shows that the cape gooseberry population used in the present study have a high level of genetic diversity with a mean value of He = 0.655, Ho = 0.431 and PIC = 0.344 using as reference the two Solanaceae genomes or the cape gooseberry root/stem transcriptome (Table 2). Our results contrast with the studies conducted by Bonilla et al. [57], where RAM markers were used in 43 cape gooseberry accessions reporting lower values of observed heterozygosity (Ho = 0.255), possibly due to the lower sample size and number of markers and their dominant nature as well as the different origin of accessions used. Our study is more consistent with the recently published report by Garzón et al. [20] on cape gooseberry diversity using 47 accessions analyzed by COSII and IRG markers, where they found a mean value of He = 0.30, Ho = 0.48, and PIC = 0.24. Considering that several accessions shared the same origin of the ones used in this study; the subtle differences in values may be due to differences in sample size, number and type of markers used. Our results indicate that the cape gooseberry is a heterozygous species consistent with the 54 % rate of cross-pollination previously reported [58].

**Table 2:** Summary statistics of genetic diversity calculated for cape gooseberry. reference transcriptome and tomato and potato reference genomes

Reference transcriptome/genome	SNPs without common tags filter	Common tags	SNPs with filter	Proportion of heterozygous sites	Ho	He	PIC
Cape gooseberry	60,663	120,124	1,699	0.639	0.430	0.647	0.343
Tomato			1,739	0.662	0.434	0.662	0.346
Potato			1,695	0.652	0.430	0.657	0.342
Mean					0.431	0.655	0.344

**Fig. 2:**
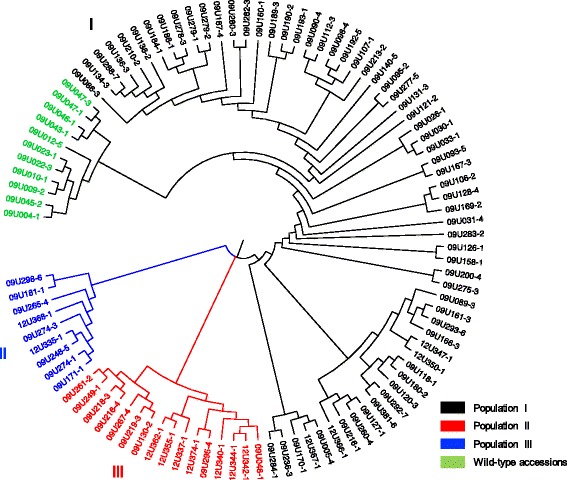
Neighbor-Joining tree based on Nei’s genetic distance of cape gooseberry SNPs. NJ-based dendrogram with cape gooseberry SNPs clustered into three subgroups. Colors correspond to each sub-population which consisted of: mostly commercial germplasm (**I**), mostly cultivated (**II**), mix of cultivated, land-races and DHs (**III**). Most of the wild-type accessions conform a subgroup (*green*) inside the sub-population I

The NJ and PCA analysis were carried out using SNPs obtained after alignment against the two reference genomes and the cape gooseberry transcriptome for confirmation of groups or sub-populations with the two analyses (Additional file 4: Figure S3, Additional file 5: Figure S4). The NJ-based dendrogram with cape gooseberry SNPs (Fig. 2) shows the collection clustered into three subgroups. Sub-population I comprised accessions from the Colombian geographic departments of Boyacá, Nariño and Cundinamarca, the main cape gooseberry producing areas in the country and DH derived materials. Sub-population II comprising mostly cultivated (commercial) accessions and Sub-population III consisted of a mixture mostly of land-races, cultivated and DHs. Inside population I there was a defined group conformed by the wild-type accessions.

The PCA analysis (Table 3) showed that the first three components explained approximate to 21.3 % of the total variation within the population for the two references genomes and the transcriptome. Zhao et al. [59] suggested that the matrices of PCA are helpful for use as a relationship matrix (Q) in association analysis, since this type of analysis is fast, has no assumptions about the population structure and gives equivalent results to those derived from computationally intensive software’s such as Structure. However, the results from this analysis showed that the matrix of PCA is not suitable for association analysis since a low percentage of the variation was captured in the first three components. According to Myles et al. [60], the problem with the estimation of Q matrices using the PCA approach, is that individuals can only vary along a few axes of differentiation that may or may not be well captured by the PCA model. Since the present study showed that there was no clear cluster structure in the first three principal components by PCA and one sub-population differed from the 3 sub-populations obtained by NJ analysis, we performed further structure analyses.

**Table 3:** Summary of the principal component analysis results using the cape gooseberry reference transcriptome and the tomato and potato reference genomes

Species	SNPs with filter	Components (%)			
		1^st^	2nd	3rd	Total
Cape gooseberry	1,699	8.8	7.3	4.5	20.6
Tomato	1,739	9.9	7.3	4.8	22.0
Potato	1,695	9.2	7.1	5.0	21.3

**Fig. 3:**
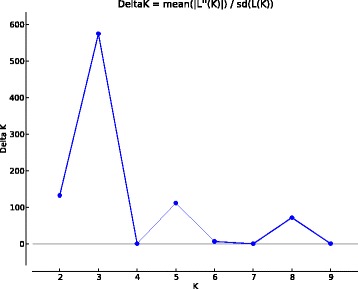
ΔK plots obtained from Evanno method derived from the SNP data. Graph of delta K values (y-axe) against assumed sub-populations (x-axe) showing the ideal number of groups present in the cape gooseberry population after use of 1,739 polymorphic SNPs. Note the highest peak for K  =  3

**Fig. 4:**
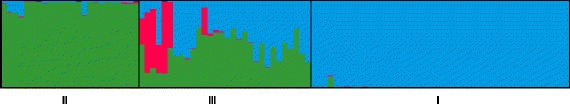
Inferred population structure of the cape gooseberry panel using the tomato SNPs matrix. STRUCTURE bar plot for K = 3 grouped by state of cultivation. Subpopulation I = Commercial germplasm, II = Cultivated, III = Mix of cultivated, land-races and DHs

Since the values of genetic diversity and clustering algorithms were similar using the two genomes and the transcriptome as reference, we chose the tomato reference genome to reduce computationally intensive analyses. Then, we selected the 1,739 polymorphic SNP markers from common tags, between tomato and cape gooseberry transcriptome, to correct for population structure to avoid spurious marker–trait associations. To find the number of sub-populations of cape gooseberry, the value based on the logarithm of probability of data likelihoods (lnP(D)) approach fluctuated continuously and never reached a plateau (data not shown). In contrast, the ΔK analysis provided by the Evanno method [39] suggested a population structure comprising of three subgroups (K = 3) (Fig. 3) with a considerably high mean F_st_ value of 0.351 indicating high population structure. These results were similar with the NJ analysis, showing three sub-populations, from which the most differentiated one was comprised of wild-type accessions (Fig. 4). These wild-type accessions provide a genetic potential for breeding programs as shown by the phenotypic severity and AUDPC values indicating potential sources of resistance responses (Table 1).

**Fig. 5:**
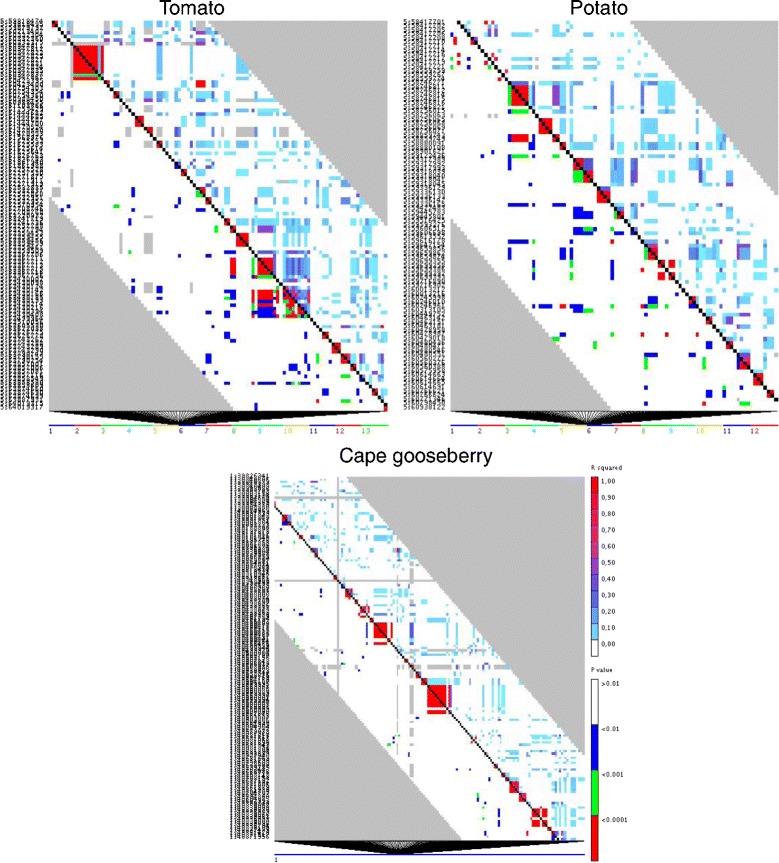
Analysis of linkage disequilibrium. Heat maps showing one region in the cape gooseberry root/stem transcriptome and a region for chromosome 6 in tomato and potato reference genomes

It was not possible to conduct an analysis of decay of LD across genetic distance in cape gooseberry because no reference genome is available. For this reason, LD was estimated using the square allele frequency correlations (r^2^) from pairs of all SNPs markers without a LD filter using the cape gooseberry transcriptome as a reference. About 4 % of total comparisons were significantly in LD with *P* ≤ 0.01 with an average r^2^ of 0.040 and a maximum value of 0.69. Similar results were found when we estimated r^2^ using tomato and potato as reference genomes (Table 4). Heat maps produced for each of the two genomes and the transcriptome showed one strong region in LD limited to chromosome 6 (Fig. 5). It would be necessary to sequence the genome of cape gooseberry in order to study thoroughly the decay of linkage disequilibrium, to more accurately identify regions of interest, and to identify recombination hot spots [61]. The whole genome sequence will be of great utility for plant breeding.

**Table 4:** Summary of the linkage disequilibrium analysis for this study

Species	SNPs without LD filter	Total comparisons	Mean r^2^ value	Comparisons in LD	Comparisons (*p* ≤ 0.01)
Cape gooseberry	60,663	1,388,575	0.040	1,316,228	54,612
Tomato	9,136	452,076	0.044	429,592	21,536
Potato	9,067	455,526	0.041	430,859	20,962

### Association analysis

The association analysis was conducted by the MLM approach with Q and K matrices fitted in the model to control spurious associations due to population structure and relatedness, respectively [42]. Using a threshold of -Log_10_ (*P*) ≥ 3, with phenotypic data from the AUDPCs and severity scales as well as high quality SNP data obtained when using the two reference genomes we did not identify any significant association after the FDR correction. This could be the result of the reduced sample size of the association mapping population along with its large heterozygosity, as it has been reported in a study conducted by Kahn et al. [62] in *Malus* genus. Besides, It is well known that a rapid LD decay occurs in cross-pollinated species needing large sample and marker density for association studies [61]. As mentioned by Zhu et al. [63], large populations are desirable for association mapping in order to obtain a high power to detect genetic effects of moderate size, but the cost of genotyping and particularly of phenotyping can be extremely elevated. However, several studies in related species as tomato [64] and unrelated species such as barley [65], demonstrates that using small population sizes (approximately 90 genotypes) with high diversity or genotypes from different origins, as we did, was adequate to identify molecular markers associated with traits of interest.

Besides sample size and heterozygosity, it is also possible that the resistance response phenotype of cape gooseberry against *F. oxysporum* is not influenced by large effect QTLs or oligogenic trait variables that can be detected when using small sample sizes, which has been demonstrated for some traits in other species [66]. Nonetheless the present study represents the first approximation to association analysis in cape gooseberry. Further and deeper GWAS analyses would need to take into account the above-mentioned considerations as well as its genome size, that represents up to 8,12 pg of nuclear DNA, being nine times larger than the tomato and potato genomes [67].

**Fig. 6:**
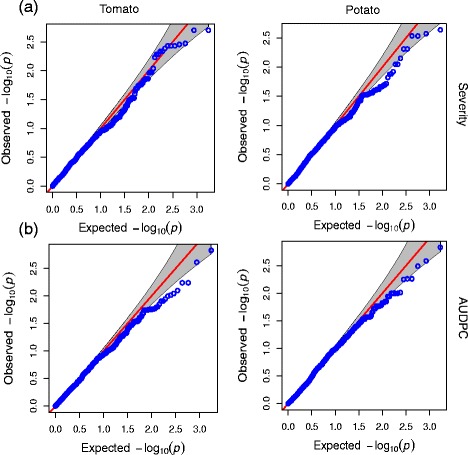
Association analysis Q-Q Plot for severity and AUDPC variables. Q-Q plots showing the ratio of the observed *p-values* (*black dots*) compared to the expected *p-value* distribution (*red lines*) for each genome for (**a**) severity, and (**b**) AUDPC

**Fig. 7:**
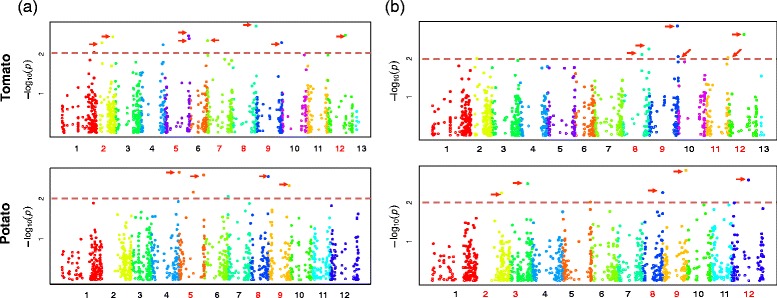
Manhattan plots of marker-trait associations for *F. oxysporum* resistance response. All -Log_10_ (*P*) > 2, observed for a data set were pooled over a GWA plot. **a** Seventeen SNPs on nine chromosomes were observed for the severity variable for both reference genomes. **b** Twelve SNPs were observed for AUDPC on 9 chromosomes for both reference genomes. The SNP markers after the FDR correction are shown by *red* arrows

Considering the aspects mentioned, we reduced the stringency threshold and analyzed Q-Q plots that supported the evidence of SNP association to the resistance response trait with lower but still significant *p*-values (*p* ≤ 0.005) before the FDR correction. In the Q-Q plots, observed *p*-values for each SNP are plotted against the values expected under the null hypothesis of no SNP associated with the trait, thus, deviations from the diagonal line suggest the SNPs markers contains values arising by a true association. Besides, the early separation of the expected *p*-values from the observed, it is due to population stratification [68] (Fig. 6). Accordingly, the MLM is performing well for accounting the population structure and familial relatedness for correcting spurious associations, as reported in association studies of tomato populations [64]. In order to reduce the amount of false-positives, we only focused the highly significant associations detected by the MLM. Using a fewer threshold, we found 28 SNPs marker of the severity and AUDPC variables which mapped to a total of 20 tomato and potato genes, with *p*-values ≤ 0.005 (Tables 5 and 6). Manhattan plots for the two traits based on the two reference genomes are shown in Fig. 7.

**Table 5:** Summary of association analysis for the severity variable

Species	SNP	Gene where SNP is located	Chromosome	Position	MAF	Before Box-cox transformation		After Box-cox transformation	
						*p*-value	FDR Adjusted	*p*-value	FDR Adjusted
							*p*-value		*p*-value
Tomato	S8_62073300^a^	Solyc08g081990.2	8	62,073,300	0.4643	0.0016	0.7129	0.0020	0.7631
	S12_46594754	Solyc12g049500.1	12	46,594,754	0.0408	0.0021	0.7129	0.0034	0.7631
	S5_61444687	Solyc05g051900.2	5	61,444,687	0.2347	0.0028	0.7129	0.0035	0.7631
	S2_42554381	Solyc02g084920.2	2	42,554,381	0.2143	0.0036	0.7129	0.0037	0.7631
	S2_42554396			42,554,396	0.2143	0.0036	0.7129	0.0037	0.7631
	S2_42554398			42,554,398	0.2143	0.0036	0.7129	0.0037	0.7631
	S5_63359509	Solyc05g054260.2	5	63,359,509	0.0663	0.0024	0.7129	0.0041	0.7631
	S7_869940	Solyc07g006030.2	7	869,940	0.4694	0.0048	0.7129	0.0046	0.7631
	S7_869967			869,967	0.4694	0.0048	0.7129	0.0046	0.7631
	S9_65753970	Solyc09g091070.1	9	65,753,970	0.2347	0.0051	0.7129	0.0052	0.7631
	S2_14016967	Solyc02g021620.2	2	14,016,967	0.0867	0.0039	0.7129	0.0053	0.7631
Potato	S5_1237068	PGSC0003DMG400007522	5	1,237,068	0.0455	0.0008	0.8937	0.0023	0.9484
	S5_58756015	PGSC0003DMG400023316		58,756,015	0.0657	0.0012	0.8937	0.0027	0.9484
	S8_42535013	PGSC0003DMG400005498	8	42,535,013	0.4646	0.0021	0.8937	0.0029	0.9484
	S8_42534983			42,534,983	0.4646	0.0021	0.8937	0.0029	0.9484
	S9_49247242	PGSC0003DMG400037435	9	49,247,242	0.2323	0.0052	0.9096	0.0049	0.9484
	S9_49247281			49,247,281	0.2323	0.0052	0.9096	0.0049	0.9484

^a^Significant associated SNP marker showed for severity and AUDPC analyses

**Table 6:** Summary of association analysis for the AUDPC variable

Species	SNP	Gene where SNP is located	Chromosome	Position	MAF	Before box-cox transformation		After box-cox transformation	
						*p*-value	FDR adjusted	*p*-value	FDR adjusted
							*p*-value		*p*-value
Tomato	S9_65685191	Solyc09g090940.2	9	65,685,191	0.4898	0.0011	0.9616	0.0015	0.9977
	S12_35821264	Solyc12g038490.1	12	35,821,264	0.4541	0.0059	0.9616	0.0024	0.9977
	S8_62073300^a^	Solyc08g081990.2	8	62,073,300	0.4643	0.0096	0.9616	0.0058	0.9977
	S8_45545340	Solyc08g061260.2	8	45,545,340	0.3878	0.0101	0.9616	0.0079	0.9977
	S9_67526887	Solyc09g098450.2	9	67,526,887	0.3929	0.0066	0.9616	0.0090	0.9977
	S11_51389456	Solyc11g069690.1	11	51,389,456	0.0561	0.0056	0.9616	0.0097	0.9977
Potato	S9_49077916	PGSC0003DMG400046263	9	49,077,916	0.4899	0.0011	0.9493	0.0015	0.9498
	S12_35550547	Not near know genes	12	35,550,547	0.4546	0.0063	0.9493	0.0026	0.9498
	S3_37927302		3	37,927,302	0.3030	0.0058	0.9493	0.0032	0.9498
	S8_42535013	PGSC0003DMG400005498	8	42,535,013	0.4647	0.0091	0.9493	0.0055	0.9498
	S8_42534983		8	42,534,983	0.4647	0.0091	0.9493	0.0055	0.9498
	S2_52227231	PGSC0003DMG400042623	2	52,227,231	0.0202	0.0028	0.9493	0.0056	0.9498

^a^Significant associated SNP marker showed for severity and AUDPC analyses

For the severity trait, one of the SNPs was mapped to the tomato gene *Solyc08g081990.2* that is related to WD-40 repeats which are involved in protein–protein interactions and with functional roles in signal transduction, regulation of transcription to cell cycle control and hypersensitive response in the defense of plants against pathogen attack [69]. The second SNP mapped to the gene *Solyc12g049500.1* that possesses the legume lectin beta domain, probably involved in the protection against pathogens by producing lipoxygenase (LOX) that catalyzes dioxygenation reactions of polyunsaturated fatty acids (PUFAs) and the secondary conversion of hydroperoxy lipids according to Roopashree et al. [70]. LOX proteins also contribute to plant growth and development, maturation, senescence and trigger metabolic response to pathogen attack [71, 72]. The third SNP mapped to *Solyc05g051900.2* gene located on chromosome 5 that is related with proteins of the Major Facilitator Superfamily (MFS). This superfamily of proteins represents the largest known group of active secondary carriers transporting a diverse range of small solutes across membranes and obtaining the energy by chemiosmotic gradients. According to Peng et al. [73], the transporters related to MFS play critical roles in plant defense against pathogen infection by exporting toxins outside the cell to reduce their accumulation. Three additional SNP markers were located on chromosome 2 on *Solyc02g084920.2* gene that is related with a proteasome subunit beta type. Suty et al. [74] suggest that plant defense genes are related to proteasome subunits which translate elicitor signals that lead to the establishment of a Systemic Acquired Resistance (SAR) against pathogen attack. More recently García-Cano et al. [75], shows that specifically, in plants, the ubiquitin/26S proteasome system (UPS) regulates protein degradation and contributes significantly to development of a wide range of processes, including immune response, development and programmed cell death.

The remaining five SNPs were not specifically related to pathogen defense/resistance genes that may suggest new roles in defense for homologous gene regions in cape gooseberry or no roles in defense/resistance at all. One mapped on chromosome 5 at the *Solyc05g054260.2* gene which has a kinesin motor activity, responsible for transport into the cell [76]. Two mapped to chromosome 7 at the gene *Solyc07g006030.2* that is related with the protein TIF31 responsible for protein-protein interactions [77]. However, an study conducted by Panthee, 2010 [13], reported that several genes related to disease resistance to *F. oxysporum* f. sp *lycopersici* are located on chromosomes 7 and 11 of the tomato genome. Another SNP marker mapped to the *Solyc09g091070.1* gene on chromosome 9, that has a function in malate dehydrogenase processes involved in plant photosynthesis and C3 and C4 in the Calvin cycle [78]; finally, one marker mapped to the *Solyc02g021620.2* gene located in the chromosome 2 which is related to the transporter superfamily of Na/K/Cl that facilitates the transport of sodium, potassium and chloride ions from the extracellular space into the interior cell [79].

Using the potato genome as reference, six SNPs associated to the severity variable with *p*-value ≤ 0.005 (Table 5) mapped to three chromosomes. One SNP marker was located within the gene *PGSC0003DMG400007522* on chromosome 5 involved in defense/resistance to pathogens; this gene is related to the F*-*box proteins that have a critical role in the control of the degradation of cellular proteins [80]*.* Several *F-box* genes have been characterized and regulate crucially important and diverse physiological processes, such as hormonal response, embryogenesis, seed germination, seedling development, floral organogenesis, lateral root formation, leaf senescence, pathogen resistance, and abiotic stress responses [81]. One of the proteins of this family known as COI1 is involved in regulating jasmonate, hormone used by plants as a signal for pathogen defense processes and is also part of the E3 ubiquitin ligase enzyme, whose function is to make a label to send signals to the proteasome to induce the degradation of the ubiquitin protein, important for defense mechanisms processes [82, 83], while SON1 and CPR30 play key roles as negative regulators in plant defense responses to pathogens [84, 85].

The remaining five SNPs were mapped to regions not specifically related to pathogen defense/resistance genes. One of the SNPs mapped to a region on chromosome 5 very close to the gene *PGSC0003DMG400023316* reported as a conserved gene but with unknown function. Other associated markers were not within any candidate gene identified so far, although they were near genes *PGSC0003DMG400005498* and *PGSC0003DMG400037435* of chromosomes 8 and 9 respectively, with unknown function.

For the AUDPC variable which is related with the time to the onset of disease symptoms, six SNPs were associated with a *p*-value ≤ 0.01 (Table 6) and mapped to four tomato chromosomes at genes involved in defense/resistance against pathogens. One of the SNPs was located at the position 62073300 of chromosome 8 within the gene *Solyc08g081990.2*, and was also associated with the severity variable. This gene was related to the WD-40 repeats important in resistance to pathogens as explained above. This result suggests that there is a possible pleiotropic effect of this marker associated with the two response variables evaluated for resistance to *F. oxysporum*. The second marker was located at the position 45545340 of chromosome 8 at the gene *Solyc08g061260.2* which is related to a large family of transmembrane receptor proteins called G protein-coupled receptors (GPCRs) in fungi and metazoans, which receive the signal and are translated through heterotrimeric G proteins. According to Liu et al. [86], the presence of GPCRs in plants is yet to be studied thoroughly; however, heterotrimeric G proteins are involved in biological processes including immunity in plants and Gβ subunits of G proteins (β- Arabidopsis G protein subunity1 AGβ1) and Gγs (γ-sununity1 Arabidopsis G protein AGG1, AGG2, AGG3) are associated with resistance to necrotrophic pathogens such as *F. oxysporum*, *Botrytis cinerea* and *Alternaria brassicicola* [87]. A recent review by Nitta et al. [88] shows the importance of G proteins in plant defense and responses to environmental stresses.

The third SNP was located at the gene *Solyc09g098450.2* of chromosome 9 related to lipase class 3 family protein involved in plant stress responses. The alpha/beta hydrolase family contains domains of lipase class 3 and has three proteins (PAD4, EDS1 and SAG101) forming a systemic signal that functions as the main barrier against pathogens [89–91]. The fourth marker was located at position 51389456 on chromosome 11 in the gene *Solyc11g069690.1* that relates to proteins called thioredoxins, which act as antioxidants, facilitating the reduction of other proteins through thiol-disulfide exchange cysteine; also maintaining the redox homeostasis. According to Vieira Dos Santos & King [92], thioredoxin plays an important role in oxidative stress tolerance in plants. They are involved in oxidative damage avoidance by reducing the power of reductases detoxifying lipid hydroperoxides or repairing oxidized proteins. It is also believed that thioredoxin is involved in defense mechanisms for the Tobacco mosaic virus and the Cucumber mosaic virus. Sun et al. [93], determined that a protein based on thioredoxin (*NtTRXh3*) is overexpressed, reduces multiplication and pathogenicity in plants. Also the overexpression of the protein enhanced the resistance to oxidative stress [93].

The remaining two SNPs were related specifically to defense/resistance genes in tomato. The first of them is located at the *Solyc09g090940.2* gene on chromosome 9 whose function is to encode the nuclear transport factor 2 (NTF2). The last marker was located on chromosome 11 at the gene *Solyc11g069690.1* with unknown function. Using the potato genome as reference, six SNPs were found associated with *p*-value ≤ 0.006 based on the AUDPC variable (Table 6) and mapped to five different chromosomes; however, none was within or next to genes with known function.

Further research is focusing on verifying the differential expression of these candidate genes by quantitative PCR in cape gooseberry. Subsequently, the genes that correlate with the resistance response by qPCR analysis will be used to create functional variants using genetic transformation or gene silencing to validate if these genes confer resistance to vascular wilt disease in cape gooseberry.

## Conclusions

The present work represents the first association mapping study in cape gooseberry. We found high heterozygosity and population structure in the diversity panel used for association and identified promising accessions to use in breeding for resistance against *F. oxysporum*. We also identified several SNPs associated with two resistance response phenotype variables that mapped to genes directly or indirectly related to pathogen resistance/defense responses involved in protein–protein interactions, signaling pathways, oxidative stress tolerance and hypersensitive response to pathogen attack. Additionally, some SNPs were found on chromosomes 7 and 11 of tomato, where QTLs associated with disease resistance have been reported previously, thus, these QTLs need validation of the homologous regions in the cape gooseberry genome. The work provides new knowledge on candidate genes involved in the *P. peruviana* – *F. oxysporum* pathosystem as a foundation for further validation in marker-assisted selection for breeding.

### Availability of supporting data

All the supporting data are included as additional files (Additional file 1: Table S1; Additional file 2: Figure S1; Additional file 3: Figure S2; Additional file 4: Figure S3 and Additional file 5: Figure S4). Raw data of the cape gooseberry transcriptome is available at NCBI Bioproject ID No. PRJNA67621.

## Additional files

Additional file 1: Table S1. List of the germplasm used for association of the cape gooseberry resistance response to *F. oxysporum* in the present study. (XLS 50 kb) Additional file 2: Figure S1. Severity scale of the diversity panel used as mapping population in the present study. (PDF 24694 kb) Additional file 3: Figure S2. Phenotypic distribution and normalization of two variables: (a) Severity and (b) AUDPC. The histograms and normality plots shows the distribution before and after Box-Cox transformations. (PDF 150 kb) Additional file 4: Figure S3. Neighbor-Joining tree based on Nei’s genetic distance using (a) potato and (b) tomato reference genomes. (PDF 54 kb) Additional file 5: Figure S4. Principal Component Analysis of the first three components representing 21.3 % of total variation of the cape gooseberry SNP markers obtained after comparisons with the: (a) cape gooseberry transcriptome (1,699 SNPs), (b) potato reference genome (1,695 SNPs) and (c) tomato reference genome (1,739 SNPs). (PDF 73 kb)

## Abbreviations

AM: association mapping
AUDPC: area under the disease progress curve
FDR: false-discovery-rate
GBS: genotyping by sequencing
GWA: genome-wide association
LD: linkage disequilibrium
MAS: marker-assisted selection
MLM: mixed linear model
PCA: principal component analysis
QTL: quantitative trait loci
SNP: single nucleotide polymorphism

## Footnotes

1 proportion of Taxa with a genotype

2 proportion of the taxon with genotypes that are not “N” for the SNP

3 proportion of the SNP genotypes for a taxon that are not “N”.
