# Supplementary figures and images for: Association analysis for disease resistance to Fusarium oxysporum in cape gooseberry (Physalis peruviana L)
Source: BMC Genomics. 2016 Mar 18;17:248. doi: 10.1186/s12864-016-2568-7 (PMC4797340; doi:10.1186/s12864-016-2568-7)

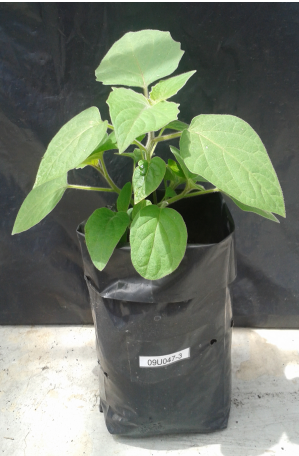

0

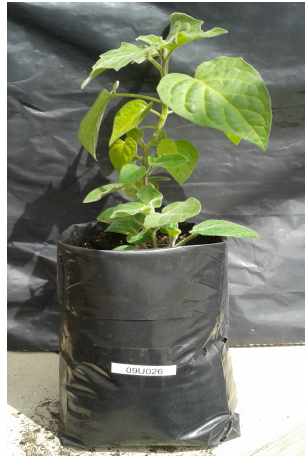

1

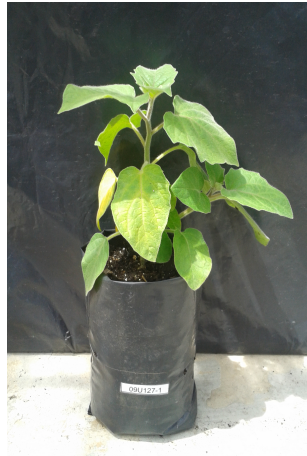

2

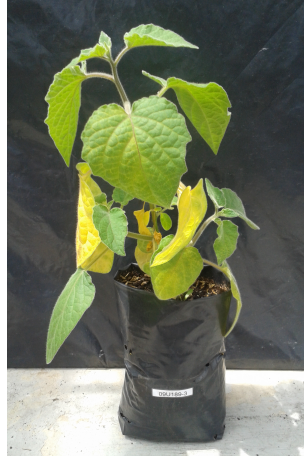

3

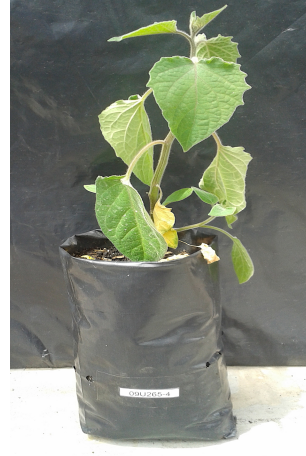

4

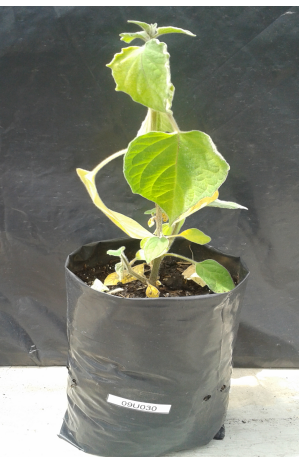

5

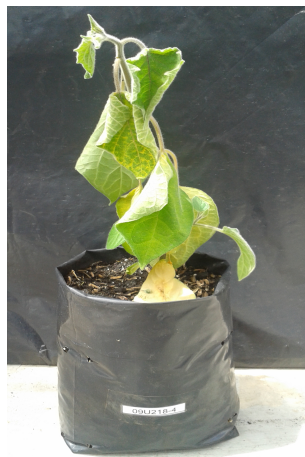

6

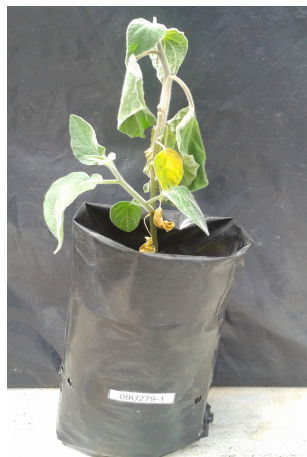

7

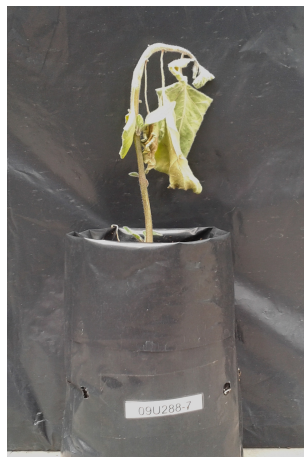

8

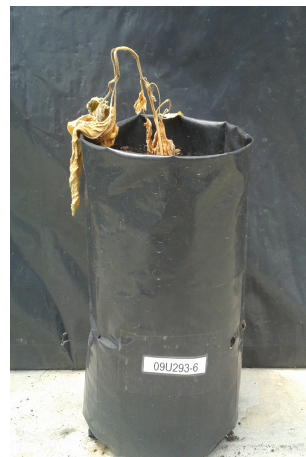

9

Supplement: Additional file 2: Figure S1. — Severity scale of the diversity panel used as mapping population in the present study. (PDF 24694 kb) [file 12864_2016_2568_MOESM2_ESM.pdf]

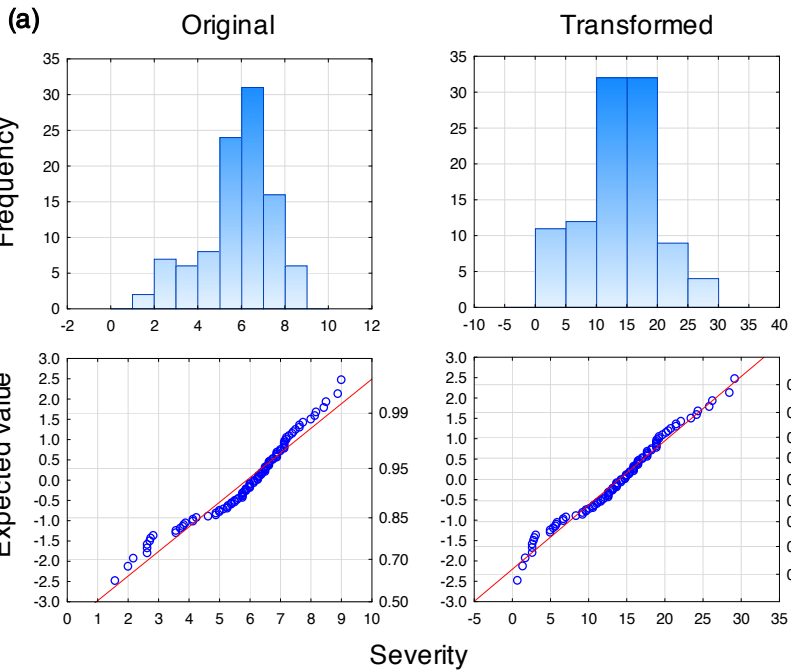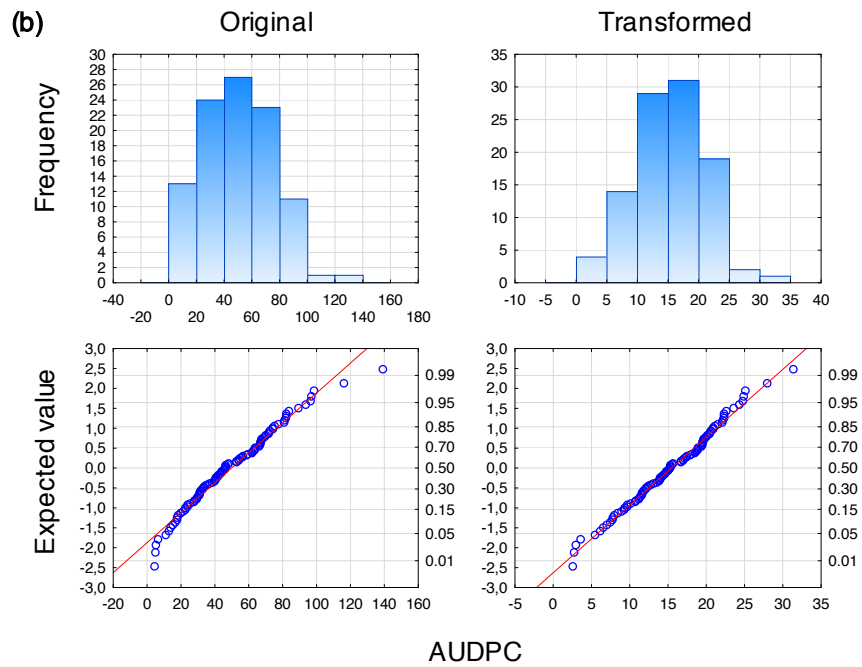

Supplement: Additional file 3: Figure S2. — Phenotypic distribution and normalization of two variables: (a) Severity and (b) AUDPC. The histograms and normality plots shows the distribution before and after Box-Cox transformations. (PDF 150 kb) [file 12864_2016_2568_MOESM3_ESM.pdf]

(a)

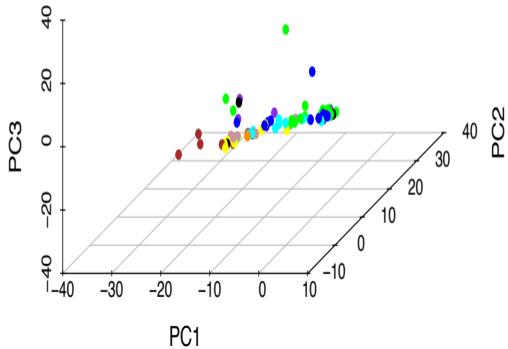

(b)

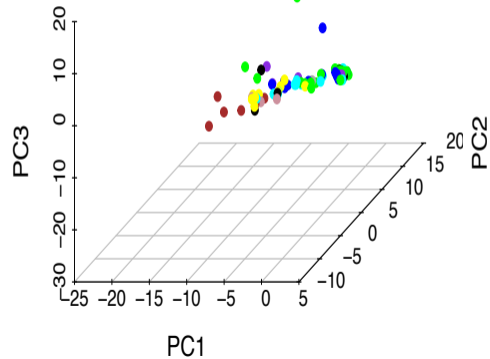

(c)

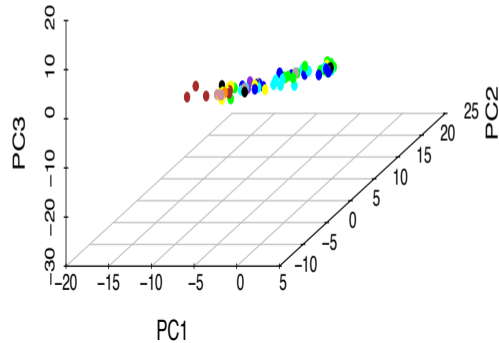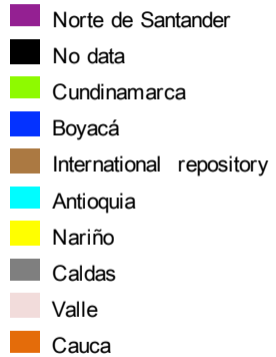

Supplement: Additional file 5: Figure S4. — Principal Component Analysis of the first three components representing 21.3 % of total variation of the cape gooseberry SNP markers obtained after comparisons with the: (a) cape gooseberry transcriptome (1,699 SNPs), (b) potato reference genome (1,695 SNPs) and (c) tomato reference genome (1,739 SNPs). (PDF 73 kb) [file 12864_2016_2568_MOESM5_ESM.pdf]
