# Supplementary material for: Association analysis for disease resistance to Fusarium oxysporum in cape gooseberry (Physalis peruviana L)
Source: BMC Genomics. 2016 Mar 18;17:248. doi: 10.1186/s12864-016-2568-7 (PMC4797340; doi:10.1186/s12864-016-2568-7)
Supplement: Additional file 4: Figure S3. — Neighbor-Joining tree based on Nei’s genetic distance using (a) potato and (b) tomato reference genomes. (PDF 54 kb) [file 12864_2016_2568_MOESM4_ESM.pdf]

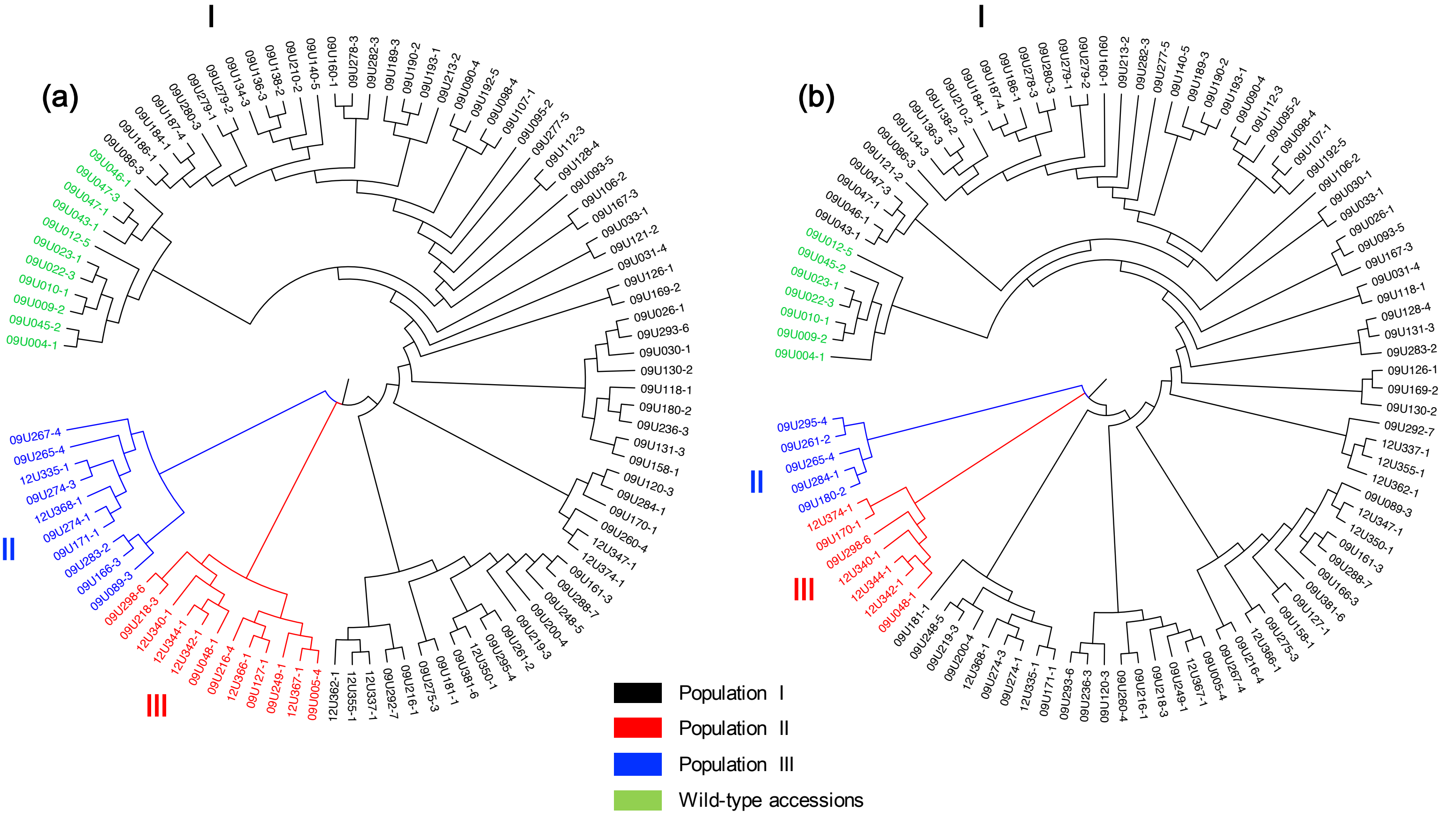
(a)
I
09U086-3
09U186-1
09U184-1
09U187-4
09U280-3
09U279-1
09U279-2
09U134-3
09U136-3
09U138-2
09U210-2
09U140-5
09U160-1
09U278-3
09U282-3
09U189-3
09U190-2
09U193-1
09U213-2
09U090-4
09U192-5
09U098-4
09U107-1
09U095-2
09U277-5
09U112-3
09U128-4
09U093-5
09U106-2
09U167-3
09U033-1
09U121-2
09U031-4
09U126-1
09U169-2
09U026-1
09U293-6
09U030-1
09U130-2
09U118-1
09U180-2
09U236-3
09U131-3
09U158-1
09U120-3
09U284-1
09U170-1
09U260-4
12U347-1
12U374-1
09U161-3
09U288-7
09U248-5
09U200-4
09U219-3
09U261-2
09U295-4
12U350-1
09U381-6
09U181-1
09U275-3
09U216-1
09U292-7
12U337-1
12U355-1
12U362-1
09U005-4
12U367-1
09U249-1
09U127-1
12U366-1
09U216-4
09U048-1
12U342-1
12U344-1
12U340-1
09U218-3
09U298-6
III
09U089-3
09U166-3
09U283-2
09U171-1
09U274-1
12U368-1
09U274-3
12U335-1
09U265-4
09U267-4
II
09U004-1
09U045-2
09U009-2
09U010-1
09U022-3
09U023-1
09U012-5
09U043-1
09U047-1
09U047-3
09U046-1
(b)
I
09U086-3
09U134-3
09U136-3
09U138-2
09U210-2
09U184-1
09U187-4
09U186-1
09U278-3
09U280-3
09U279-1
09U279-2
09U160-1
09U213-2
09U282-3
09U277-5
09U140-5
09U189-3
09U190-2
09U193-1
09U090-4
09U112-3
09U095-2
09U098-4
09U107-1
09U192-5
09U106-2
09U030-1
09U033-1
09U026-1
09U093-5
09U167-3
09U031-4
09U118-1
09U128-4
09U131-3
09U283-2
09U126-1
09U169-2
09U130-2
09U292-7
12U337-1
12U355-1
12U362-1
09U089-3
12U347-1
12U350-1
09U161-3
09U288-7
09U166-3
09U381-6
09U127-1
09U158-1
09U275-3
12U366-1
09U216-4
09U267-4
09U005-4
12U367-1
09U249-1
09U218-3
09U216-1
09U260-4
09U120-3
09U236-3
09U293-6
09U171-1
12U335-1
09U274-1
09U274-3
12U368-1
09U200-4
09U219-3
09U248-5
09U181-1
09U048-1
12U342-1
12U344-1
12U340-1
09U298-6
09U170-1
12U374-1
III
09U180-2
09U284-1
09U265-4
09U261-2
09U295-4
II
09U121-2
09U047-3
09U047-1
09U046-1
09U043-1
09U012-5
09U045-2
09U023-1
09U022-3
09U010-1
09U009-2
09U004-1
Population I
Population II
Population III
Wild-type accessions
